# Multidisciplinary quantitative and qualitative assessment of IDH-mutant gliomas with full diagnostic deep learning image reconstruction

**DOI:** 10.1016/j.ejro.2024.100617

**Published:** 2024-12-04

**Authors:** Christer Ruff, Paula Bombach, Constantin Roder, Eliane Weinbrenner, Christoph Artzner, Leonie Zerweck, Frank Paulsen, Till-Karsten Hauser, Ulrike Ernemann, Georg Gohla

**Affiliations:** a Department of Diagnostic and Interventional Neuroradiology, Eberhard Karls-University Tuebingen, Tuebingen D-72076, Germany; b Department of Neurology and Interdisciplinary Neuro-Oncology, University Hospital Tuebingen, Tuebingen D-72076, Germany; c Hertie Institute for Clinical Brain Research, Eberhard Karls University Tuebingen Center of Neuro-Oncology, Tuebingen D-72076, Germany; d Center for Neuro-Oncology, Comprehensive Cancer Center Tuebingen-Stuttgart, University Hospital of Tuebingen, Eberhard Karls University of Tuebingen, Tuebingen D-72070, Germany; e Department of Neurosurgery, University of Tuebingen, Tuebingen D-72076, Germany; f Department of Diagnostic and Interventional Radiology, Diakonie Klinikum Stuttgart, Stuttgart D-70176, Germany; g Department of Radiation Oncology, University Hospital Tuebingen, Tuebingen D-72076, Germany

**Keywords:** IDH-mutant gliomas, Magnetic resonance imaging, Deep learning, Image reconstruction, Multidisciplinary, Diagnostic accuracy, Visual perception preference

## Abstract

**Rationale and Objectives:** Diagnostic accuracy and therapeutic decision-making for IDH-mutant gliomas in tumor board reviews are based on MRI and multidisciplinary interactions.

**Materials and Methods:**

This study explores the feasibility of deep learning-based reconstruction (DLR) in MRI for IDH-mutant gliomas. The research utilizes a multidisciplinary approach, engaging neuroradiologists, neurosurgeons, neuro-oncologists, and radiotherapists to evaluate qualitative aspects of DLR and conventional reconstructed (CR) sequences. Furthermore, quantitative image quality and tumor volumes according to Response Assessment in Neuro-Oncology (RANO) 2.0 standards were assessed.

**Results:**

All DLR sequences consistently outperformed CR sequences (median of 4 for all) in qualitative image quality across all raters (p < 0.001 for all) and revealed higher SNR and CNR values (p < 0.001 for all). Preference for all DLR over CR was overwhelming, with ratings of 84 % from the neuroradiologist, 100 % from the neurosurgeon, 92 % from the neuro-oncologist, and 84 % from the radiation oncologist. The RANO 2.0 compliant measurements showed no significant difference between the CR and DRL sequences (p = 0.142).

**Conclusion:**

This study demonstrates the clinical feasibility of DLR in MR imaging of IDH-mutant gliomas, with significant time savings of 29.6 % on average and non-inferior image quality to CR. DLR sequences received strong multidisciplinary preference, underscoring their potential for enhancing neuro-oncological decision-making and suitability for clinical implementation.

## 1 Introduction

Astrocytoma and oligodendrogliomas are types of adult diffuse gliomas associated with mutations in isocitrate dehydrogenase (IDH). In accordance with the 2021 WHO classification, IDH-mutant astrocytoma is classified as grade 2, 3, or 4, based on both histological and molecular criteria, notably the absence of 1p19q codeletion, which confirms an astrocytoma rather than an oligodendroglioma [1]. Incidence rates per 100,000 people are 0.88 for astrocytoma and 0.34 for oligodendroglioma (including the subgroups formerly referred to as anaplastic, which is no longer used) [2].

Patients with gliomas rely heavily on MRI for diagnosis, treatment planning in radiotherapy and neurosurgery, and continuous monitoring [3]. Essential MRI protocols include T1-weighted contrast-enhanced and T2-weighted sequences, often featuring fluid-attenuated inversion recovery (FLAIR) imaging [4]. The aforementioned tumor types show different imaging characteristics depending on the subtypes. Imaging characteristics vary by tumor subtype: grade 2 astrocytoma typically show no contrast enhancement, while grades 3 and 4 often do [5]. Oligodendroglioma, on the other hand, may display uneven contrast enhancement, and the appearance of new enhancement in a previously non-enhancing, untreated tumor may be indicative of malignant transformation [6]. T2-weighted imaging is essential for diagnostic work-up and assessment of tumor extension and infiltration [6]. In addition, T2-weighted imaging plays a pivotal role in the evaluation of treatment effects in the follow-up of these tumors, which can be challenging [7].

Conventional MRI techniques are constrained by the trade-off between high-resolution imaging and scan time. A critical consideration is the optimal balance between image resolution, signal-to-noise ratio (SNR), and acquisition time. These three imaging parameters have a complex relationship: higher resolution allows for observation of smaller details, but typically reduces SNR and/or increases the required imaging time [8]. Concurrently, a minimum level of SNR is necessary to differentiate the signal of interest from system noise. Additionally, the scan time should be kept to a minimum, as MR imaging resources are scarce, costly, and prolonged scan times are uncomfortable for the patient, leading to motion artifacts in the images [9]. While traditional MRI is an invaluable tool for glioma imaging, it is not without limitations that impact its diagnostic precision. The precise delineation of tumor boundaries, an essential element in the effective planning and monitoring of treatment, can be adversely affected by the presence of image quality issues. Additionally, traditional MRI images are susceptible to the introduction of artifacts and noise, which can obscure critical details and complicate the assessment of tumor heterogeneity, a key factor in glioma grading and treatment response. To address these challenges, established methods including parallel acquisition techniques (PATs) and compressed sensing (CS) to speed up the examination time were applied [10], [11]. Nevertheless, it has been demonstrated that PATs lead to a reduction in SNR that is proportional to the square root of the PAT factor, while CS may generate images that appear overly smooth [12], [13], [14].

In order to overcome these obstacles, the application of artificial intelligence (AI) in medical imaging has garnered significant interest [15]. This has resulted in significant improvements in image quality and the incorporation of neural network technologies [16]. In particular, deep learning (DL) has emerged as a crucial technique for surpassing the limitations of traditional acceleration methods in imaging. State-of-the-art DL models are designed to enhance clinical diagnostics by improving accuracy, speed, and overall capabilities [17]. Models such as deep neural networks, variational autoencoders, and generative adversarial networks accelerate MRI acquisition, reducing patient discomfort and improving workflow [17]. Convolutional neural networks (CNNs) with attention mechanisms and U-net architectures enhance MRI spatial resolution for better anatomical visualization, while autoencoders and CNNs with residual connections reduce noise in low SNR scenarios, improving image quality and diagnostic confidence [18]. These models also mitigate common MRI artifacts, enhancing diagnostic accuracy.

In the field of brain tumor management, AI has been shown to have significant potential in a range of applications, including diagnosis, prognosis, and treatment planning beyond accelerating and enhancing MRI [19], [20]. The incorporation of AI tools into radiological and pathological workflows holds promise for advancing neuro-oncology [21], [22]. DL algorithms have played a pivotal role in ensuring spatial consistency, correcting artifacts, and eliminating distortions in MRI images, thereby enabling more precise tumor localization and segmentation [23]. DL models frequently achieve optimized performance in specific cohorts, as generalized models often struggle with variations in data quality and patient characteristics across diverse populations [17], [24], [25]. For glioblastomas, DL algorithms have already achieved a 30 % improvement in acquisition time as well as an improvement in subjective image quality [9]. However, there are no DL implementations including a multidisciplinary evaluation for the more diverse group of IDH-mutant gliomas to date.

As DL-based MRI reconstruction techniques prove effective in research, they are likely to see increased clinical adoption. AI-based techniques demonstrated to significantly enhance the subjective image quality of neuroradiological images, including FLAIR sequences, T2-weighted, and T1-weighted images, thereby boosting diagnostic confidence [9], [26], [27], [28]. Nevertheless, this improved image impression should be viewed with circumspection, as AI-generated images may yield disparate outputs contingent on the underlying network. Consequently, it is challenging to ascertain their veracity. However, these studies have primarily focused on the evaluations by (neuro)radiologists. Nevertheless, a multidisciplinary approach is essential for assessing the precision and efficacy of diagnosis, surveillance, and interdisciplinary treatment planning. Specifically, MRI images are vital for radiotherapists to visualize anatomy for segmentation of the target and organs at risk, aid in dose calculation and optimization, and serve as a reference for positioning during treatment. They are equally important for neurosurgeons for surgical planning and navigation [29], [30].

In light of the lack of DL implementations for IDH-mutant gliomas, the absence of objective image quality parameters, and the deficiency in multidisciplinary approaches, we have initiated a systematic comparative study to assess the diagnostic efficacy of conventionally reconstructed (CR) versus deep learning reconstructed (DLR) sequences within a fully diagnostic glioma MRI protocol. This protocol includes T1-weighted, T2-weighted turbo spin echo (TSE) sequences, and FLAIR imaging for IDH-mutant gliomas. We hypothesized that the evaluated DLR MR images would provide improved quantitative and qualitative image quality not only for neuroradiologists, but also for neurosurgeons, neuro-oncologists, and radiotherapists. Furthermore, it was postulated that DLR images would be preferred for imaging assessment by the various disciplines.

## 2 Materials and methods

### 2.1 Study design

**Fig. 1:**
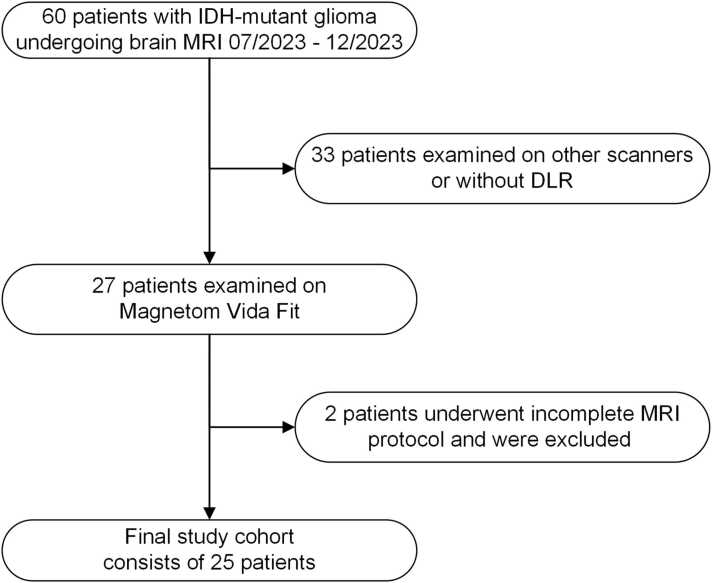
Study flowchart and patient enrollment.

This presented monocentric retrospective study was approved by the institutional review board under code 761/2023B02 and is conducted in accordance with the Declaration of Helsinki and its subsequent amendments. It encompasses 25 IDH-mutant glioma patients who underwent cerebral MRI for therapy evaluation or initial diagnosis between July and December 2023. Exclusion criteria were patients with duplicates, scans from 1.5 Tesla MRI machines, artifacts caused by external materials, individuals under 18 years, and those with not completely acquired DL or CR MRI datasets (Fig. 1).

### 2.2 Imaging acquisition and deep learning reconstruction algorithm

All examinations were conducted using a single 3-Tesla clinical MRI scanner (MAGNETOM Vida Fit, Siemens Healthineers, Erlangen, Germany). Patients were examined in a supine position with a 32-channel head coil. The standard glioma protocol for the institution is in accordance with the consensus recommendations for standardized brain tumor imaging [31] and consists of the following sequences: FLAIR in axial plane, T1-weighted TSE imaging in axial plane with and without contrast medium, T1-weighted three-dimension isotropic magnetization-prepared rapid gradient-echo imaging in sagittal plane with and without contrast medium, T2-weighted TSE imaging in axial and coronal plane, diffusion-weighted imaging with two different acquired b-values in axial plane (0 s/mm², 1000 s/mm²), and corresponding apparent diffusion coefficient mapping, Dynamic Susceptibility Contrast (DSC) perfusion imaging using a flow rate of 3 ml/s followed by a saline flush of 20 ml (0.1 mmol/kg body weight gadobutrol; Gadovist, Bayer Healthcare, Leverkusen, Germany). The axial images were obtained with a slice thickness of 4 mm, while the coronal images were obtained with a slice thickness of 3 mm. The undersampled DLR sequences were acquired and reconstructed separately for FLAIR, T1-weighted contrast-enhanced, and T2-weighted TSE imaging immediately after the corresponding conventional sequence type with the same planes and slice thickness. For conventional reconstructed axial FLAIR (FLAIR_CR_), axial T1-weighted contrast-enhanced (T1CE_CR_), and axial T2-weighted imaging (T2_CR_) the acceleration factor was set to two phase-encoding (PE) steps, while for the DLR sequences it was set to four PE steps: axial FLAIR (FLAIR_DLR_), axial T1-weighted contrast-enhanced (T1CE_DLR_), and axial T2-weighted imaging (T2_DLR_). The DLR were conducted offline by using the accessible standard clinical hardware infrastructure of the MRI workstation with no discernible time impact on the clinical workflow. The in-plane resolution was enhanced and, in accelerated sequences, typically doubled when compared to CR sequences, with simultaneous reduction in acquisition times. Table 1 presents a comprehensive overview of the acquisition parameters for these sequences and their DLR versions, showcasing an enhanced in-plane resolution. The selection of the FLAIR-, T2-, and T1-weighted contrast-enhanced images was based on the sequences required for the Response Assessment in Neuro-Oncology (RANO) 2.0 compliant measurements.

**Table 1:** MRI acquisition parameters.

**Parameters**	**FLAIR**_**CR**_	**FLAIR**_**DLR**_	**T2**_**CR**_	**T2**_**DLR**_	**T1CE**_**CR**_	**T1CE**_**DLR**_
**Field of view (mm)**	230	230	230	230	230	230
**Voxel size (mm)**	0.4 × 0.4 × 4.0	0.4 × 0.4 × 4.0	0.3 × 0.3 × 4.0	0.3 × 0.3 × 4.0	0.4 × 0.4 × 4.0	0.4 × 0.4 × 4.0
**Slice thickness (mm)**	4	4	4	4	4	4
**Number of slices**	40	40	40	40	40	40
**Base resolution**	320	320	384	384	304	304
**Parallel imaging factor**	2	4	2	4	2	4
**Acceleration mode**	GRAPPA	GRAPPA	GRAPPA	GRAPPA	GRAPPA	GRAPPA
**Reference lines**	72	72	72	72	72	72
**TR (ms)**	8800	8800	4220	4220	2170	2170
**TE (ms)**	81	81	82	82	9.7	9.7
**Averages**	1	1	1	1	1	1
**Concatenations**	2	2	2	2	2	2
**Acquisition time (min)**	2:40	1:57	1:09	0:51	2:03	1:19

FLAIR = Fluid-Attenuated Inversion Recovery; CR = conventional reconstruction; DLR = deep learning reconstruction; CE = contrast-enhanced; GRAPPA = GeneRalized Autocalibrating Partial Parallel Acquisition (parallel imaging technique); TR = repetition time; TE = echo time.

The acquisition time for the FLAIR_CR_ was 2:40 min but that for the FLAIR_DLR_ was 1:57 min. For T1-weighted TSE imaging, the T1CE_CR_ acquisition time was 2:03 min, while for the T1CE_DLR_ sequence was 1:19 min. For T2-weighted TSE imaging, the T2_CR_ acquisition time was 1:09 min but T2_DLR_ required 0:51 min.

The U.S. Food and Drug Administration (FDA)-approved DLR algorithm used in this study adheres to the methodology described in detail in a previous study by Herrmann et al. and has also been used in other studies [9], [32]. The image reconstruction algorithm offers two options: a fixed iterative reconstruction method or a variational network approach [33], [34]. In this study, the deep neural network model combines physical principles with data-driven techniques for MRI. The system employs a fixed unrolled algorithm comprising numerous cascades, which integrate data consistency and CNN-based regularization. The regularization model is structured in a hierarchical fashion with an iterative network, which improves memory efficiency by adjusting the resolution of feature maps. The CNN module, termed the "Deep, Iterative, Hierarchical Network," expands the Down-Up network design via a hierarchical block arrangement. In its processing of undersampled k-space data and coil sensitivity maps, the algorithm employs a separate acquisition to obtain a bias field for the purpose of image homogenization. The network was trained using over 25000 slices acquired from volunteer scans performed on various clinical 1.5 T and 3 T MAGNETOM scanners (Siemens Healthcare, Erlangen, Germany). Subsequently, the algorithm was incorporated into the scanner's reconstruction pipeline with the aim of investigating its potential clinical applications.

### 2.3 Qualitative image quality analysis

The evaluation team consisted of four experienced raters: a neuroradiologist (Rater 1) with nine years of experience, a neurosurgeon (Rater 2) with fifteen years, a neuro-oncologist (Rater 3) with ten years, and a radiation oncologist (Rater 4) with twenty-nine years of experience. The raters independently assessed 25 image datasets, including CR and DLR images featuring axial FLAIR, axial T2-weighted TSE, and axial T1-weighted contrast-enhanced TSE imaging. The evaluations were conducted in a randomized order. Raters were blinded to the reconstruction type, clinical and radiological reports, and each other's assessments, with all patient- or sequence-identifying markers eradicated to ensure anonymity. The study readings were performed in certified reading room conditions on a dedicated workstation (GE Centricity PACS RA 1000, version 7.0.2; General Electric (GE) Healthcare, Chicago, Illinois, USA) and certified diagnostic radiology monitors (RadiForce RX350, Eizo Corporation, Hakusan, Ishikawa, Japan). The datasets were evaluated by the four raters based on multiple parameters using a detailed Likert scale from 1 to 5 with 5 being the best to ensure sufficient image quality gradation. The evaluation criteria for the 25 image datasets included image quality, tumor conspicuity (delineation of the tumor), and diagnostic confidence.

The following score was used to qualitatively evaluate the overall image quality: 1 = non-diagnostic images, 2 = poor image quality (significant blurring, noise, or artifacts that hinder interpretation but some structures are still visible), 3 = fair image quality (noticeable blurring, noise, or artifacts that moderately affect interpretation), 4 = good image quality (minimal blurring, noise, or artifacts, allowing for effective interpretation), and 5 = excellent image quality (exceptionally clear with no blurring, noise, or artifacts, providing optimal conditions for interpretation).

Tumor conspicuity was rated with the following score: 1 = very poor conspicuity (the tumor is almost indistinguishable from the surrounding tissue, making identification very difficult), 2 = poor conspicuity (the tumor is faintly visible but blends significantly with the surrounding tissue, leading to uncertainty in identification), 3 = fair conspicuity (the tumor is moderately visible but could be confused with surrounding structures, requiring careful analysis), 4 = good conspicuity (the tumor is clearly visible and distinguishable from the surrounding tissue with minimal effort), and 5 = excellent conspicuity (the tumor stands out prominently and is easily distinguishable from the surrounding tissue, ensuring confident identification).

The degree of diagnostic confidence was evaluated with the following scheme: 1 = non-diagnostic images, 2 = low diagnostic confidence (confidence in the diagnosis is low, with significant doubt about the findings and interpretation), 3 = moderate diagnostic confidence (there is moderate confidence in the diagnosis, with some uncertainty that requires further verification), 4 = high diagnostic confidence (there is high confidence in the diagnosis, with clear findings and minimal doubt), and 5 = excellent confidence (there is very high confidence in the diagnosis, with clear, definitive findings and no doubt).

Each score incrementally reflected increasing levels of approval or quality, allowing for a nuanced assessment of the images.

In addition, two board-certified neuroradiologists, Rater 1 and an additional neuroradiologist with three years of experience, independently examined the data sets for artifacts, focusing on pulsation artifacts and artificial distortions due to DLR technology. These assessments of both conventional and accelerated datasets were conducted in a random and mixed order across separate sessions, with at least a two-week washout period between each session to mitigate bias. Artifacts were ranked on a Likert scale from 1 to 5, where a score of 1 indicated severe artifacts significantly distorting the image, and a score of 5 represented a complete absence of artifacts.

In a follow-up session, Raters 1 and 5 conducted unblinded evaluations of both CR and DLR sequences to specifically compare the DLR sequences for any artificial incidental findings, using the conventional sequences as a reference for ground truth. This comparison aimed to directly assess the impact of DLR technology on image fidelity.

### 2.4 Rater preference

In the preference analysis, each of the four experienced raters independently reviewed the 25 image datasets after a 2-week period following their initial analysis. These datasets included FLAIR, T2-weighted TSE, and T1-weighted TSE sequences, evaluated on a per-patient basis. The raters were tasked with indicating their preferred sequence reconstruction type (CR vs. DLR) for both individual sequence evaluations and an overall assessment of the entire dataset. They were unaware to the patients' clinical information and the specific imaging techniques used for each sequence.

### 2.5 Quantitative image quality assessment

A quantitative assessment of image quality was conducted by measuring the signal intensity in the region of interest (ROI). The SNR and contrast-to-noise ratio (CNR) were measured in a joint, unblinded analysis by two neuroradiologists. This approach was employed to ensure the reproducibility of the measurements and to prevent potential measurement errors. The average signal intensity of non-tumorous white matter (S_WM_), and the tumors' region of interest (S_L_) were selected for solid parts or, when not detectable, in treatment-related changes. Areas of necrotic, cystic, and hemorrhagic components were avoided. An area of homogeneous signal was selected for measurement. The standard deviation (SD) of the SI in the background ROI was considered as a measure of noise (SD_noise_). This ROI had a standardized size of 300 mm^2^ for background noise and 20 mm^2^ for S_L_ and S_wm_, respectively. In parallel imaging, the distribution of noise across the image is not uniform. [35]. Consequently, a precise global estimation is unfeasible, as it would require a measurement in a homogeneous region outside of the object of interest, which is not a viable approach. While an exact measurement of noise across the entire image is unworkable, a localized estimation of the noise can be attained. Accordingly, in the present study, we conducted local noise measurements in a region proximate to the target signal measurement site, as previously recommended by Heverhagen [36]. The SNR and CNR of the lesions were calculated using the following equations: SNR = $\frac{\mathrm{SL}}{\mathrm{SDnoise}}$, CNR = $\frac{\left(\mathrm{SL}-\mathrm{Swm}\right)}{\mathrm{SDnoise}}$.

### 2.6 RANO 2.0

In 2023, the RANO Working Group published updated response criteria for gliomas [37]. The RANO 2.0 criteria propose a standard set of criteria for both high- and low-grade gliomas to be used for all trials, irrespective of the treatment modalities being evaluated. In addition to the aforementioned guidance, response criteria for contrast-enhancing tumors, non–contrast-enhancing tumors, and tumors with both enhancing and non-enhancing components are proposed. This is intended to improve the assessment of response and progression in glial tumors. In this study, a board-certified neuroradiologist with 9 years of experience and with a background in multicenter and therapy studies performed the bidimensional RANO-compliant measurements of the 25 image datasets for both CR and DLR sequences. The measurements were conducted using dedicated software solutions (mint Lesion, version 3.9.2; Mint Medical GmbH, Heidelberg, Germany). The rater was unaware of the type of reconstruction. The RANO 2.0 compliant measurements encompassed the identification of non-enhancing non-target lesions (NENT) through the utilization of FLAIR sequences, the discernment of enhancing target lesions (ET) with a minimum dimension of 1.0 × 1.0 cm through axial T1-weighted contrast-enhanced TSE images, and the delineation of enhancing non-target lesions (ENT) < 1.0 cm through axial T1-weighted contrast-enhanced TSE images. The area based on the short and long axis as well as the volume of the lesions was quantified by the used software. On the slice of the maximum tumor extension, the long and short axes were indicated for the evaluation of a target or non-target lesion. The volume of the tumor lesions is a critical determinant of the tumor response assessment in accordance with the RANO 2.0 criteria. For the final RANO statement of complete response, partial response, stable disease, or progression, a consensus reading was conducted by two board-certified neuroradiologists with 8 and 9 years of experience, based on the aforementioned measurements and access to previous examinations including the baseline.

### 2.7 Statistical analysis

Statistical analysis was conducted using SPSS Statistics Version 28 (IBM, Armonk, New York, USA). Continuous variables were presented as mean ± standard deviation (SD) and ordinal variables as median and interquartile range (IQR). Image quality comparisons between groups utilized the Wilcoxon signed-rank test. Interrater reliability was evaluated using the Kendall coefficient of concordance (W). Kendall W is calculated by contrasting the number of concordant and discordant pairs, yielding a value between −1 and 1, where 0 suggests no correlation, 1 indicates perfect agreement, and −1 denotes perfect disagreement. The effect sizes for Kendall W were categorized as follows: none (0 to < 0.10), fair (0.10 to < 0.30), moderate (0.30 to < 0.50), large (0.50 to < 0.70), and substantially large to almost perfect agreement (0.70–1.00). For RANO and SNR and CNR comparisons between CR and DLR sequences paired sample t-tests were used. The significance level was set at alpha = 0.05.

## 3 Results

This retrospective study included 25 consecutive patients who underwent MRI for suspected or confirmed IDH-mutant glioma, with all tumors histopathologically verified. The average patient age was 56 ± 12.5 years, encompassing 17 males and 8 females, ranging from 21 to 72 years old. The average duration since initial diagnosis was 57 ± 59.9 months. Notably, 60 % (15/25) of the patients experienced structural epilepsy. Comprehensive clinical data were available for all participants at the time of the imaging study. The patient characteristics are shown in Table 2.

**Table 2:** Patient characteristics.

**Characteristics**	**Values**
Number of patients	n = 25
Age, mean ± Standard Deviation (years)	56 ± 12.5
Sex (male vs. female)	n = 17 (68 %) vs. n = 8 (32 %)
Initial diagnosis without previous therapy	n = 2 (8 %)
Time of imaging since first diagnosis, (mean ± Standard Deviation (SD, months)	57 ± 59.9
**Tumor type**	
Astrocytoma grade 2	10
Astrocytoma grade 3	2
Astrocytoma grade 4	4
Oligodendroglioma	9
**Clinical Scores**	
Karnofsky Performance Scale Index (KPS in %), median [interquartile range]	90 [80−100]
ECOG Performance Status Scale,	
median [interquartile range]	0 [0−1]
Neurologic Assessment in Neuro-Oncology (NANO), median [interquartile range]	0 [0−1]
Montreal Cognitive Assessment (MoCA)*,	
median [interquartile range]	29.5 [27.75–30]
Mini-Mental State Examination (MMSE)*, median [interquartile range]	30 [29 - 30]
Distress thermometer (DT)*, median [interquartile range]	2.5 [0.25–5]
**These 25 patients received the following therapy:**	
Surgery	n = 22/25 (88 %)
Radiotherapy	n = 19/25 (76 %)
Bevacizumab	n = 2/25 (8 %)
Temozolomide	n = 13/25 (52 %)
PCV scheme	n = 3/25 (12 %)
Lomustine	n = 2/25 (8 %)

* MOCA was determined in 14/25 patients, MMSE in 14/25 patients and DT in 24/25 patients. ECOG = Eastern Cooperative Oncology Group; PCV = procarbazine, lomustine, and vincristine.

The implementation of DLR techniques resulted in significant reductions in MRI acquisition times across all evaluated sequences, despite having a higher in-plane resolution. Specifically, the FLAIR-weighted sequence demonstrated a time savings of 26.9 %, while the T2-weighted sequence achieved a reduction of 26.1 %. Notably, the T1-weighted contrast-enhanced sequence exhibited the greatest improvement, with a 35.8 % reduction in acquisition time compared to conventionally reconstructed sequences.

### 3.1 Image quality-based analysis

Qualitative image quality was consistently rated higher in DLR imaging compared to CR imaging across all sequences by all raters (all p ≤ 0.002).

**Fig. 2:**
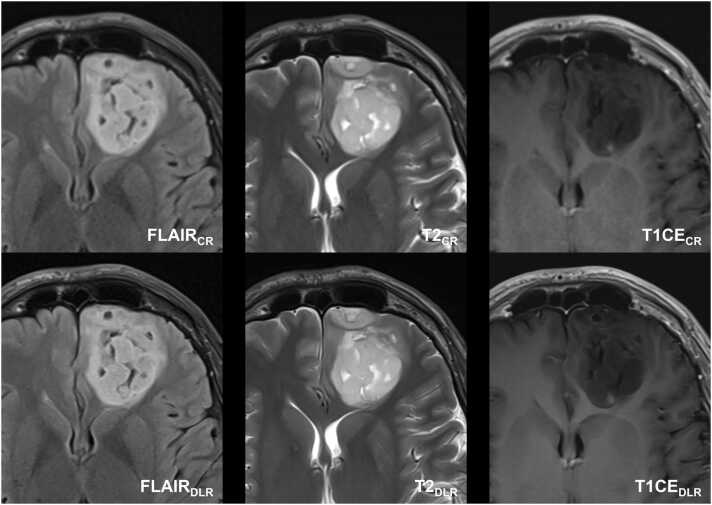
Thirty-six-year-old man with histologically confirmed oligodendroglioma in the left frontal lobe with O6-Methylguanine-DNA-methyltransferase (MGMT) promoter methylation. Deep learning reconstructed (DLR) fluid-attenuated inversion recovery images (FLAIR), T2-weighted images, and T1-weighted contrast-enhanced (CE) images showed non-inferior image quality, image sharpness, and tumor conspicuity as well as less image noise compared to conventional reconstruction (CR). Furthermore, DLR images demonstrate less image noise.

**Fig. 3:**
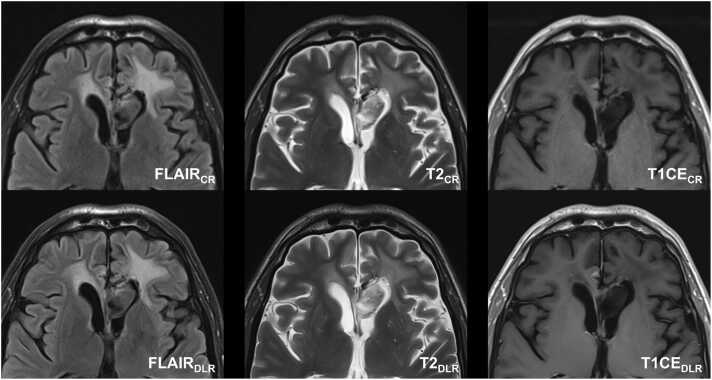
Follow-up MRI of a fifty-nine-year-old man after radiation therapy and current therapy with Bevacizumab of a left frontal histologically confirmed astrocytoma grade 2. Deep learning reconstructed (DLR) fluid-attenuated inversion recovery images (FLAIR), T2-weighted images, and T1-weighted contrast-enhanced (CE) images revealed sharper and better delineation of the intra-ventricular tumor structures in the DLR T2-weighted images compared to the standard image technique. Note the DLR algorithm's tendency to produce images of the tumor's internal structure that appear more uniform and softer in contrast to conventionally reconstructed (CR) images. Note minimal (banding) artifacts in T2- and T1-weighted DLR images.

**Fig. 4:**
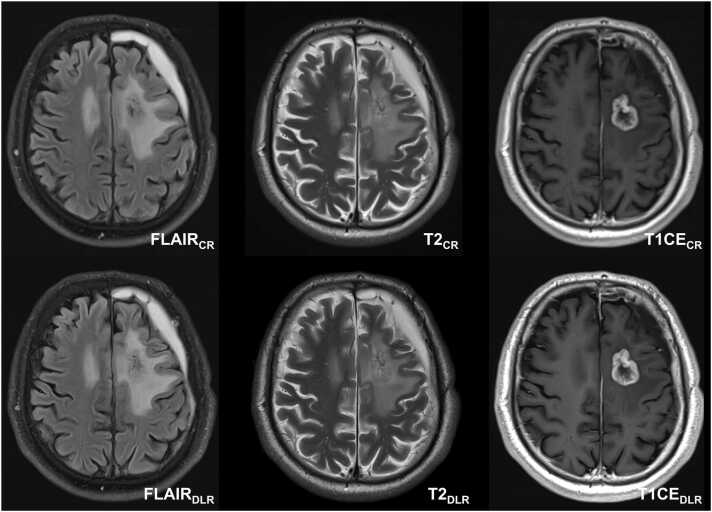
Follow-up MRI of an astrocytoma grade 4 without O6-Methylguanine-DNA-methyltransferase (MGMT) promoter methylation in the left frontal lobe in a fifty-eight-year-old man after partial resection, radiation therapy, and temozolomide. The accelerated deep learning reconstructed (DLR) images presented non-inferior overall image quality and preferred visual perception compared to conventional reconstructed images (CR). CR: conventional reconstruction; DLR: deep learning reconstructed technique; FLAIR = fluid-attenuated inversion recovery images; CE = contrast-enhanced.

For tumor conspicuity, rater 2 (neurosurgeon) evaluated FLAIR and T2-weighted DLR sequences with no significant difference to the CR images (p = 0.102, respectively). Furthermore, rater 3 (neuro-oncologist) also voted T1CE with no statistically significant difference between DLR and CR images (p = 0.059). For all other raters and sequence types, DLR showed non-inferior tumor conspicuity compared to the corresponding CR images (all p ≤ 0.020). Fig. 2, Fig. 3, Fig. 4 illustrate the non-inferior overall subjective image quality, image sharpness, tumor conspicuity, and image noise of DLR images in comparison to CR images for FLAIR-weighted, T2-weighted, and T1-weighted contrast-enhanced axial series in IDH-mutant gliomas. In addition, Fig. 3 shows an example of an intraventricular tumor structure that is perceived as sharper and better delineated in T2-weighted DLR images compared to T2-weighted CR images.

The high diagnostic confidence levels observed in the DLR and CR images did not exhibit any notable differences when compared across the FLAIR and T2-weighted imaging datasets (all p ≥ 0.083). Analyses of T1CE showed also no significant differences between DLR and CR of rater 1, 3, and 4 (all p ≥ 0.317). In contrast, rater 2 revealed higher diagnostic confidence by using the DLR version of the T1CE sequence compared to CR (p = 0.005).

For image artifacts, there were no significant differences in artifacts between CR and DLR sequences (p ≥ 0.05), except for T1CE imaging, where Rater 1 found fewer artifacts in DLR images (p = 0.011). Findings from both neuroradiologists concerning artifacts can be found in Supplementary Table 1. Fig. 2 depicts minimal banding artifacts in T2-weighted and T1-weighted contrast-enhanced DLR images.

The analysis of the qualitative image quality, tumor conspicuity, and diagnostic confidence conducted by the multidisciplinary team is presented in detail in Table 3.

**Table 3:** Multidisciplinary rating of qualitative image quality, tumor conspicuity, and diagnostic confidence of deep learning reconstructed images and conventional recorded images by four experienced raters. Raters consist of a neuroradiologist (Rater 1), a neurosurgeon (Rater 2), a neuro-oncologist (Rater 3), and a radiation oncologist (Rater 4). The p-values were calculated using the Wilcoxon signed-rank test.

			**CR**		**DLR**		
			Mdn (IQR)	M ± SD	Mdn (IQR)	M ± SD	p-value
Image quality	FLAIR	Rater 1	4 (4−4)	4.08 ± 0.28	5 (5−5)	4.96 ± 0.28	< 0.001
		Rater 2	5 (4−5)	4.48 ± 0.59	5 (5−5)	4.92 ± 0.28	0.002
		Rater 3	4 (4−4)	3.80 ± 0.41	5 (4−5)	4.72 ± 0.46	< 0.001
		Rater 4	4 (4−4)	3.92 ± 0.49	5 (4−5)	4.56 ± 0.51	< 0.001
	T2	Rater 1	4 (4−4)	4.20 ± 0.41	5 (5−5)	4.88 ± 0.32	< 0.001
		Rater 2	4 (4−5)	4.36 ± 0.57	5 (5−5)	4.96 ± 0.28	< 0.001
		Rater 3	4 (4−5)	4.36 ± 0.57	5 (5−5)	4.92 ± 0.27	< 0.001
		Rater 4	4 (4−4)	3.80 ± 0.41	5 (4−5)	4.48 ± 0.59	< 0.001
	T1CE	Rater 1	4 (4−4)	3.92 ± 0.28	5 (5−5)	4.96 ± 0.20	< 0.001
		Rater 2	4 (4−4)	4.08 ± 0.28	5 (5−5)	4.92 ± 0.27	< 0.001
		Rater 3	4 (3−4)	3.72 ± 0.46	5 (4−5)	4.68 ± 0.48	< 0.001
		Rater 4	4 (3.5–4)	3.76 ± 0.44	5 (4−5)	4.52 ± 0.50	< 0.001
Tumor conspicuity	FLAIR	Rater 1	4 (4−4)	4.12 ± 0.32	5 (5−5)	4.96 ± 0.20	< 0.001
		Rater 2	5 (4−5)	4.56 ± 0.50	5 (5−5)	4.80 ± 0.40	0.102
		Rater 3	4 (3−4)	3.64 ± 0.57	4 (4−4)	4.08 ± 0.40	< 0.001
		Rater 4	4 (3−4)	3.72 ± 0.68	4 (3−5)	4.08 ± 0.81	0.003
	T2	Rater 1	4 (4−5)	4.48 ± 0.50	5 (5−5)	4.84 ± 0.37	0.020
		Rater 2	5 (4−5)	4.64 ± 0.48	5 (5−5)	4.80 ± 0.40	0.102
		Rater 3	4 (3−4)	3.64 ± 0.64	4 (4−4)	4.00 ± 0.58	0.003
		Rater 4	4 (3−4)	3.64 ± 0.64	4 (4−4)	4.00 ± 0.57	0.003
	T1CE	Rater 1	4 (4−4)	4.08 ± 0.28	5 (5−5)	4.92 ± 0.27	< 0.001
		Rater 2	4 (4−5)	4.44 ± 0.50	5 (4.5–5)	4.76 ± 0.43	0.011
		Rater 3	3 (3.5–4)	3.80 ± 0.50	4 (4−4)	4.00 ± 0.50	0.059
		Rater 4	3 (3−3)	3.08 ± 0.57	4 (3−4)	3.60 ± 0.50	< 0.001
Diagnostic confidence	FLAIR	Rater 1	5 (5−5)	4.92 ± 0.28	5 (5−5)	4.92 ± 0.27	1
		Rater 2	5 (4−5)	4.64 ± 0.48	5 (4.5–5)	4.76 ± 0.43	0.083
		Rater 3	4 (3−4)	3.72 ± 0.46	4 (4−4)	3.80 ± 0.41	0.317
		Rater 4	4 (4−4)	3.96 ± 0.45	4 (4−4)	3.96 ± 0.46	1
	T2	Rater 1	5 (5−5)	4.80 ± 0.41	5 (5−5)	4.84 ± 0.37	0.317
		Rater 2	5 (4−5)	4.72 ± 0.46	5 (5−5)	4.80 ± 0.40	0.317
		Rater 3	4 (3−4)	3.64 ± 0.49	4 (3−4)	3.72 ± 0.46	0.157
		Rater 4	4 (3−4)	3.72 ± 0.46	4 (3.5–4)	3.76 ± 0.44	0.317
	T1CE	Rater 1	5 (4−5)	4.72 ± 0.46	5 (4.5–5)	4.76 ± 0.44	0.317
		Rater 2	4 (4−5)	4.48 ± 0.50	5 (5−5)	4.80 ± 0.41	0.005
		Rater 3	4 (3.5–4)	3.80 ± 0.50	4 (4−4)	3.88 ± 0.53	0.317
		Rater 4	4 (4−4)	3.96 ± 0.46	4 (4−4)	3.92 ± 0.57	0.564

CR = conventional reconstruction; DLR = deep learning reconstruction; Mdn = median; IQR = interquartile range; M = mean; SD = standard deviation; FLAIR = Fluid-Attenuated Inversion Recovery; T2 = T2-weighted images; T1CE = T1-weighted contrast-enhanced images.

### 3.2 Interrater intraprotocol agreement of image quality-based analysis

The interrater intraprotocol agreement (CR vs. CR) for overall image quality was fair to moderate, with Kendall W values ranging from 0.196 to 0.394. The interrater intraprotocol agreement (DLR vs. DLR) was moderate with Kendall W values ranging from 0.354 to 0.416.

The interrater intraprotocol agreement (CR vs. CR) for tumor conspicuity was moderate to substantially large, with Kendall W values ranging from 0.357 to 0.858. The interrater intraprotocol agreement (DLR vs. DLR) was large to substantially large with Kendall W values ranging from 0.513 to 0.914.

Similar results are displayed for the interrater intraprotocol agreement for diagnostic confidence both for CR (ranging from 0.422 to 0.868) as well as DLR protocol (ranging from 0.547 to 0.858). Table 4 delineates the interrater intraprotocol agreement results for Kendall W.

**Table 4:** Mean (range) interrater intraprotocol agreement between raters using Kendall W.

	**Kendall W**
Overall image quality_CR_	0.303 (0.196–0.394)
FLAIR_CR_	0.320
T2_CR_	0.394
T1CE_CR_	0.196
Overall image quality_DLR_	0.393 (0.354–0.416)
FLAIR_DLR_	0.354
T2_DLR_	0.408
T1CE_DLR_	0.416
Tumor conspicuity_CR_	0.634 (0.357–0.858)
FLAIR_CR_	0.357
T2_CR_	0.689
T1CE_CR_	0.858
Tumor conspicuity_DLR_	0.701 (0.513–0.914)
FLAIR_DLR_	0.780
T2_DLR_	0.858
T1CE_DLR_	0.547
Diagnostic confidence_CR_	0.672 (0.422–.868)
FLAIR_CR_	0.728
T2_CR_	0.868
T1CE_CR_	0.422
Diagnostic confidence_DLR_	0.728 (0.547–0.858)
FLAIR_DLR_	0.780
T2_DLR_	0.858
T1CE_DLR_	0.547

FLAIR = Fluid-Attenuated Inversion Recovery; CR = conventional reconstruction; DLR = deep learning reconstruction; CE = contrast-enhanced.

### 3.3 Rater preference between DLR and conventionally reconstructed images

**Fig. 5:**
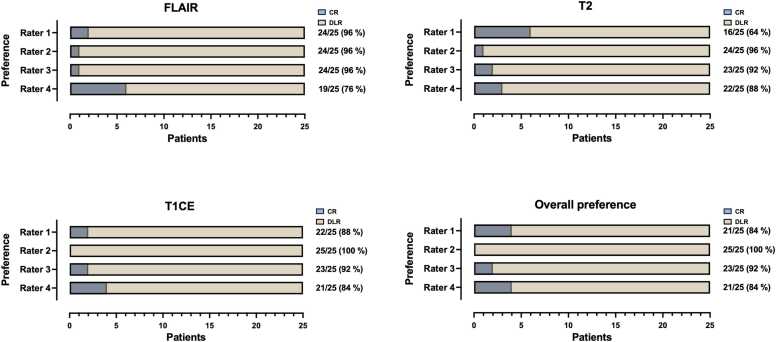
Visual preferences in the review of 25 paired magnetic resonance image sets containing astrocytoma grade 2 – 4 and oligodendroglioma by four experienced raters. Raters consist of a neuroradiologist (Rater 1), a neurosurgeon (Rater 2), a neuro-oncologist (Rater 3), and a radiation oncologist (Rater 4). CR = conventional reconstruction; DLR = deep learning reconstructed technique; FLAIR = fluid-attenuated inversion recovery images; T2 = T2-weighted images; T1CE = T1-weighted contrast-enhanced images.

All four raters of the multidisciplinary team consisting of a neuroradiologist (rater 1), a neurosurgeon (rater 2), a neuro-oncologist (rater 3), and a radiation oncologist (rater 4) preferred DLR over CR images as a whole image set (84 %, 100 %, 92 %, and 84 %, respectively), as shown in Fig. 5. In detail, all raters preferred FLAIR_DLR_ over FLAIR_CR_ (96 %, 96 %, 96 %, and 76 % respectively), T2_DLR_ over T2_CR_ (64 %, 96 %, 92 %, and 88 % respectively), and T1CE_DLR_ over T1CE_CR_ (88 %, 100 %, 92 %, and 84 % respectively). Fig. 4 illustrates an exemplary case with a left frontal IDH-mutant glioma following resection, radiation, and temozolomide with increased preference for the DLR images.

### 3.4 Quantitative image quality assessment

In the quantitative analysis, the SNR showed significant improvements with the use of DLR. Specifically, SNR for DLR images increased from 112.61 ± 36.67–187.84 ± 50.57 (p < 0.001) for the FLAIR sequences, from 232.40 ± 110.41–350.69 ± 159.37 (p < 0.001) for the in T2-weighted TSE sequences, and from 135.38 ± 86.44–320.46 ± 165.59 (p < 0.001) for the T1-weighted contrast-enhanced sequences compared to CR images. Similarly, the CNR between the S_L_ and S_WM_ also increased for DLR compared to CR images: in FLAIR from 44.37 ± 26.14–71.52 ± 42.51 (p < 0.001), in T2-weighted TSE from 73.27 ± 14.65–115.94 ± 23.19 (p < 0.001), and in T1-weighted contrast-enhanced imaging from 57.22 ± 12.2–114.27 ± 24.36 (p < 0.001). Detailed results are documented in Table 5.

**Table 5:** Quantitative analysis between conventionally reconstructed and deep learning reconstructed MRI sequences.

	FLAIR_CR_	FLAIR_DLR_	p-value	T2_CR_	T2_DLR_	p-value	T1CE_CR_	T1CE_DLR_	p-value
SNR	112.61 ± 36.67	187.84 ± 50.57	< 0.001	232.40 ± 110.41	350.69 ± 159.37	< 0.001	135.38 ± 86.44	320.46 ± 165.59	< 0.001
CNR	44.37 ± 26.14	71.52 ± 42.51	< 0.001	73.27 ± 14.65	115.94 ± 23.19	< 0.001	57.22 ± 12.20	114.27 ± 24.36	< 0.001

Data are presented as mean ± standard deviation. FLAIR = Fluid-Attenuated Inversion Recovery; SNR = signal-to-noise ratio; CNR = contrast-to-noise ratio; CR = conventional reconstruction; DLR = deep learning reconstruction; CE = contrast-enhanced.

### 3.5 RANO 2.0

**Fig. 6:**
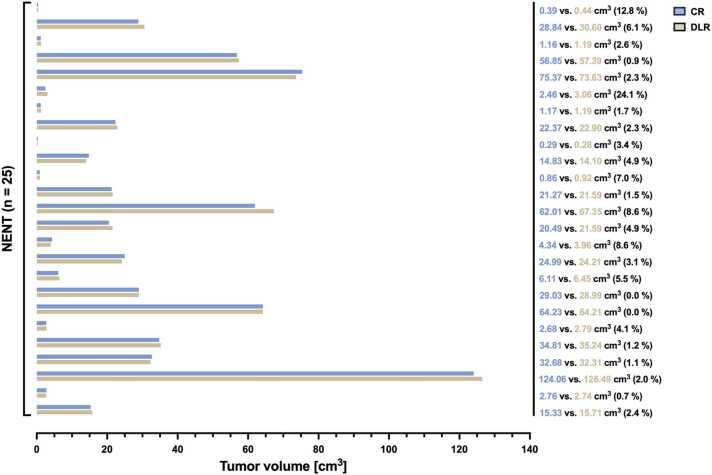
RANO-compliant measurements of non-enhancing non-target lesions (NENT) through the utilization of conventional reconstructed (CR) and deep learning reconstructed (DLR) FLAIR sequences of 25 paired image sets. FLAIR = fluid-attenuated inversion recovery images.

In no case was a different RANO 2.0 statement found amongst the standard and DLR sequences. Similarly, no differentiation was noted in classification (ET vs. ENT) for small lesions. Furthermore, no significant differences were observed in lesion size measurements between the CR and DLR sequences, irrespective of tumor size in each patient. Specifically, the volume of NENT (n = 25) was 28.96 ± 31.47 cm^3^ for FLAIR_CR_ compared to 29.45 ± 32.01 cm^3^ for FLAIR_DLR_ with a p-value of 0.142, indicating no significant difference. The paired differences between DLR and CR RANO-compliant NENT lesion measurements are illustrated in Fig. 6. The volume of the two ET lesions was 1.03 vs. 1.08 cm^3^ (Δ = 4.9 %) and 15.36 vs. 15.48 cm^3^ (Δ = 0.8 %) for T1CE_CR_ compared to T1CE_DLR_. Statistical significance was not calculated due to the small sample size (n = 2). One ENT lesion was measured with a volume of 0.95 cm^3^ in the T1CE_CR_ compared to 0.93 cm^3^ (Δ = 2.1 %) in the T1CE_DLR_ sequence.

### 3.6 Pooled analysis of qualitative image quality assessment and rater preference

**Fig. 7:**
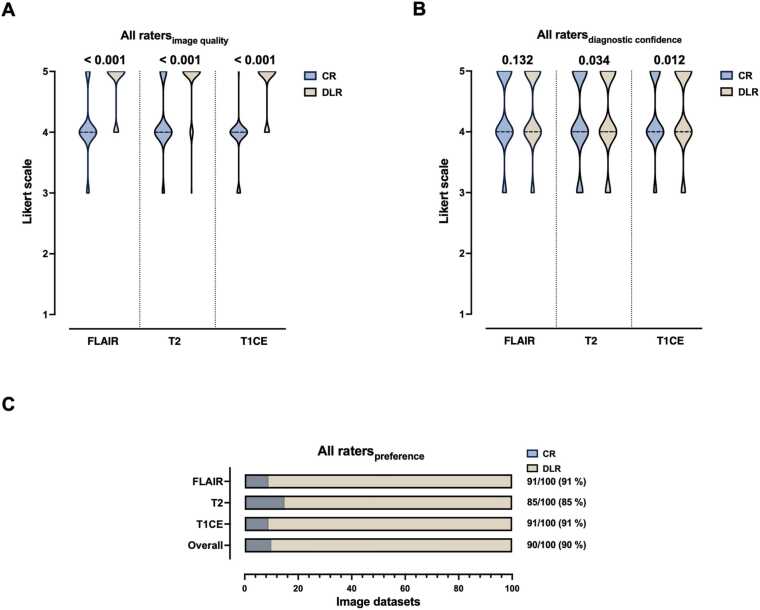
Multidisciplinary rating of image quality (A) and diagnostic confidence (B) of deep learning reconstructed images and conventional recorded images by four experienced raters in a pooled data analysis. Likert scale ranging from 1 to 5 with 5 being the best rating. Significant differences are indicated. The dotted lines represent the median. (C) displays the visual preferences of the four 25 paired magnetic resonance image sets containing astrocytoma grade 2 – 4 and oligodendroglioma of all raters in a pooled data analysis. CR = conventional reconstruction; DLR = deep learning reconstructed technique; FLAIR = fluid-attenuated inversion recovery images; T2 = T2-weighted images; T1CE = T1-weighted contrast-enhanced images.

Image quality was consistently rated higher in DLR imaging compared to CR imaging across all sequences by all raters in a pooled data analysis (all p < 0.001), as shown in Fig. 7A. Regarding diagnostic confidence, there was no significant difference between CR and DLR imaging for FLAIR (p = 0.132), but significant differences for T2 (p = 0.034) and for T1CE (p = 0.012) in favor of DLR, Fig. 7B. A more detailed insight into the results, including mean (standard deviation) in addition to median (interquartile ranges), can be found in Supplementary Table 2.

In a pooled data analysis, raters of the multidisciplinary team indicated a preference for DLR over CR images as a whole image set (90 %). In detail, raters preferred FLAIRDLR over FLAIRCR (91 %), T2DLR over T2CR (85 %), and T1CEDLR over T1CECR (91 %). These findings are illustrated in Fig. 7C.

## 4 Discussion

DLR is increasingly finding its way into everyday clinical practice. However, experience in real-world clinical settings of this new type of reconstruction in direct comparison with CR MRI sequences is not yet sufficient and is under development. Oncological MRI is an integral component of a comprehensive multidisciplinary approach that encompasses the evaluation of therapeutic efficacy and related therapeutic decisions. Neuro-oncological imaging, in particular, is characterized by a high degree of interdisciplinarity in the context of increasing therapeutic possibilities with new immunotherapies, targeted therapies, and vaccinations. Previous studies on the qualitative and diagnostic performance of novel DL MRI have focused exclusively on radiologists, which does not reflect the necessary multidisciplinarity in neuro-oncological imaging. The successful integration of DL technology into routine clinical practice depends on the acceptance of various specialties who are convinced that DLR techniques are not inferior to CR techniques. The integration of interdisciplinary research and collaboration between radiologists and other specialties has the potential to yield artificial techniques that are more closely aligned with clinical requirements. This approach effectively bridges the gap between theoretical knowledge and practical application. Such collaborative endeavors also foster a collective sense of ownership and responsibility, thereby facilitating the adoption and optimization of artificial intelligence tools in clinical settings. Consequently, this study investigated the assessment of qualitative image quality by neuroradiologists, neurosurgeons, neurooncologists, and radiotherapists in IDH-mutant gliomas and evaluated the specialty-dependent subjective image impressions and preferences.

Our study revealed a reduction in acquisition time by 35.8 % for T1-weighted contrast-enhanced, by 26.9 % for FLAIR-weighted, and by 26.1 % for T2-weighted imaging for DLR sequences compared to the CR image in IDH-mutant gliomas without compromising subjective image quality or diagnostic confidence. This is consistent with previous studies of the DLR mechanism in other neuroradiological and non-neurological areas [9], [28], [38], [39]. Furthermore, DLR MRI consistently achieves higher subjective and objective image quality Likert scores than CR sequences in IDH-mutant gliomas regardless of the medical specialty. This finding is consistent with previous research on DLR in neuro-oncology imaging as well as non-neuroradiological tumor fields by radiologists [9], [27], [38], [40], [41], [42].

Furthermore, tumor conspicuity in DLR images was rated with higher subjective Likert scores than those of CR. Additionally, our analysis showed no significant differences in diagnostic confidence between the two protocols, except for one outlier. This consistency aligns with earlier studies across various neuroradiological and abdominopelvic assessments, which found that DLR typically maintains or improves diagnostic confidence [9], [43], [44], [45].

Notably, there is a variation in the level of assessment for tumor conspicuity and diagnostic confidence between raters with and without extensive daily imaging experience, observed in both CR and DLR sequences. Lee et al. showed that, depending on their level of knowledge and the frequency with which they use imaging in their daily routine, radiologists also prefer DLR images to varying degrees due to the different levels of sharpness and the unfamiliar image impression among other things [46]. These could be the reasons why in our study, all raters demonstrated a consistent preference for DLR over CR images across all sequence types in most cases. Given that not only radiologists but also other specialties rely on imaging on a daily basis for therapy decisions, the implications of this finding are significant. Among the raters, the neurosurgeon and the neuro-oncologist exhibited the strongest preference for DLR images. The raters noted that tumors were more clearly delineated using DLR, particularly in FLAIR sequences, with a subjectively better distinction between tumor and edema. The neurosurgeon also appreciated the clearer demarcation of smaller contrast-enhancing structures and the subjectively improved contrast in T1-weighted sequences in DLR images. When the neuro-oncologist preferred CR images, it was typically in cases with ambiguous lesions or when it was challenging to distinguish imaging features from artifacts, possibly due to less familiarity with regular imaging practices. The radiation oncologist demonstrated the strongest preference for FLAIR_CR_ images among all raters. Accurately defining target volumes and identifying organs at risk are critical in this context. While the sharpness and enhanced clarity of DLR images aid in delineating target areas, the radiation oncologist sometimes favored CR images when tumors appeared different from their usual depiction or when DLR introduced unfamiliar artifacts. These results must be interpreted with caution, as they reflect the subjective opinions of individual researchers and do not represent the consensus of the entire specialist group. Despite this limitation, the differences between the disciplines are noteworthy and warrant further investigation.

In T2-weighted TSE imaging, there is a significant variation in evaluations among the raters, particularly when comparing the assessments of the neuroradiologist with those of the neuro-oncologist and radiation oncologist. This discrepancy could be due to multiple factors. Prior research has highlighted that DLR images exhibit lower levels of image noise compared to images obtained through traditional full-scan techniques, a trait that can make DLR images seem unnatural to seasoned radiologists [12], [47]. The DLR algorithm tends to produce images where the internal structure of the tumor appears more uniform and has a softer contrast compared to conventional images. Additionally, individual preferences and the level of familiarity with the types of images commonly seen in regular practice also play a role in these differing evaluations.

The qualitative image quality rating is supported by the quantitative assessment of SNR and CNR, which is significantly better for DLR compared to CR. This is in line with other studies that have also shown improved SNR and CNR for DLR images [48], [49], [50], [51].

The impact of DLR on tumor extent evaluation is crucial for understanding the comparability and interchangeability of different reconstruction types in daily clinical practice. When assessing tumors using RANO 2.0 criteria, our study found no significant differences in the size and volume measurements of ET, ENT, and NENT lesions. This consistency aligns with findings from previous studies, which also reported no variation in lesion size measurements between CR and DLR T2-weighted sequences in glioma and abdominopelvic imaging for ensuring reliable tumor sizing and subsequent response assessment [9], [44], [52]. However, it is important to recognize that there is no universally accepted imaging standard that definitively determines the true size of tumors.

There are various potential advantages of the study such as the assessment from a diverse group of medical professionals. This multidisciplinary approach ensures that the findings are relevant and applicable across various specialties involved in neurooncological imaging and decision-making. The planning and implementation of cancer treatments by surgical, medical, and radiation oncologists is contingent upon the utilization of diverse imaging modalities for diagnostic purposes [53]. It is evident that no single cancer subspecialist can function in isolation. The integration of services is a crucial aspect of optimizing treatment outcomes, minimizing complications, and positively influencing long-term results. The study suggests that DLR MRI can be integrated into routine clinical practice, potentially improving the efficiency and effectiveness of neuro-oncological imaging protocols by reducing scan time while enhancing image quality and maintaining or improving diagnostic confidence as was shown by earlier research [9]. Combining quantitative metrics with qualitative evaluations provides a robust and comprehensive understanding of the imaging technique.

However, this study is subject to several disadvantages and limitations. Firstly, it involved a relatively small cohort of 25 patients, which was determined by power analysis. However, future multicenter studies with larger cohorts are needed for comprehensive validation and to perform subgroup analysis. Secondly, by focusing solely on IDH-mutant gliomas and excluding other types of brain tumors, there is a risk of selection bias, which may limit the applicability of the results to other brain tumor subgroups. However, as suggested by prominent review articles on DL, this study concentrated on assessing the performance of DL within a specific cohort [17], [24], [25]. Furthermore, it is important to highlight that CR images were set as the ground truth and no external ground truth as histopathological data were available. Third, the inclusion of diverse age groups and genders introduces potential confounding variables that could influence the outcomes, although this diversity reflects the clinical routine at a university hospital. Fourth, qualitative assessments were conducted exclusively by a multidisciplinary team of experienced raters familiar with CR images. The study did not include evaluations by inexperienced raters or comparisons between experienced and inexperienced raters, which could have provided additional insights. Qualitative evaluations are inherently susceptible to individual biases, potentially affecting the reliability of the findings. Fifth, our study included only one neuroradiologist, neurosurgeon, neuro-oncologist, and radiation oncologist. Further studies with more specialists from these medical disciplines are therefore necessary to mitigate individual tendencies. Sixth, further investigations regarding image artifacts and the partially different impression of tumor structures between images reconstructed with deep learning and conventionally recorded images are necessary to examine the specific features of these novel sequences in detail. Seventh, CR and DLR sequences were acquired sequentially, one immediately after the other, which is currently necessary when setting CR as the ground truth. However, this approach introduces a risk of non-identical image alignment, particularly in cases where patient motion occurs between acquisitions. Lastly, the study was limited to MRI scans from a single scanner by one manufacturer, restricting the generalizability of the results to other settings.

In conclusion, this study demonstrated the clinical feasibility of DL in the MR-imaging of IDH-mutant gliomas with a substantial reduction in acquisition time of 29.6 % on average. FLAIR_DLR_, T2_DLR_, and T1CE_DLR_ consistently achieved improved qualitative and quantitative image quality Likert scores compared to CR sequences. Furthermore, our multidisciplinary approach indicates that not only neuroradiologists but also neurosurgeons, neuro-oncologists, and radiotherapists involved in the glioma treatment and assessment of treatment response preferred DLR over CR images. The strong preference for DLR sequences among all raters underscores the potential for these advanced imaging techniques to improve diagnostic and therapeutic decision-making in neuro-oncological practice and to promote acceptance of this approach for implementation in clinical routine.

## CRediT authorship contribution statement

**Leonie Zerweck:** Writing – review & editing, Validation, Methodology, Formal analysis. **Frank Paulsen:** Writing – review & editing, Validation, Methodology, Investigation, Formal analysis, Conceptualization. **Till-Karsten Hauser:** Writing – review & editing, Methodology, Investigation, Formal analysis, Data curation, Conceptualization. **Ulrike Ernemann:** Writing – review & editing, Supervision, Resources, Project administration. **Paula Bombach:** Writing – review & editing, Validation, Investigation, Data curation. **Constantin Roder:** Writing – review & editing, Validation, Methodology, Investigation. **Eliane Weinbrenner:** Writing – review & editing, Validation, Methodology. **Christoph Artzner:** Writing – review & editing, Validation, Formal analysis. **Georg Gohla:** Writing – review & editing, Writing – original draft, Visualization, Validation, Supervision, Investigation, Formal analysis, Data curation, Conceptualization. **Christer Ruff:** Writing – review & editing, Writing – original draft, Visualization, Investigation, Formal analysis, Conceptualization.

## Declaration of Competing Interest

The authors declare that they have no known competing financial interests or personal relationships that could have appeared to influence the work reported in this paper.
