# Supplementary material for: Multidisciplinary quantitative and qualitative assessment of IDH-mutant gliomas with full diagnostic deep learning image reconstruction
Source: Eur J Radiol Open. 2024 Dec 4;13:100617. doi: 10.1016/j.ejro.2024.100617 (PMC11664152; doi:10.1016/j.ejro.2024.100617)
Supplement: Supplementary file 1 — Supplementary material. [file mmc1.docx]

**Supplementary Table 1.** Median (interquartile range) artifact ratings on a 5 point Likert scale in conventionally reconstructed and deep learning reconstructed sequences.

|  | FLAIR_CR_ | FLAIR_DLR_ | p-value  FLAIR_CR vs._  FLAIR_DLR_ | T2_CR_ | T2_DLR_ | p-value  T2_CR vs._  T2_DLR_ | T1CE_CR_ | T1CE_DLR_ | p-value  T1CE_CR vs._ T1CE_DLR_ |
| --- | --- | --- | --- | --- | --- | --- | --- | --- | --- |
| Artifacts_Rater 1_ | 5 [5 – 5] | 5 [4.5 – 5] | 0.059 | 5 [4 – 5] | 4 [4 – 5] | 0.090 | 4 [3 – 4] | 4 [4 – 4] | 0.011 |
| Artifacts_Rater 5_ | 5 [5 – 5] | 5 [5 – 5] | 0.083 | 5 [4 – 5] | 5 [4 – 5] | 0.248 | 4 [3 – 4] | 4 [4 – 4] | 0.132 |

FLAIR = Fluid-Attenuated Inversion Recovery; CR = conventional reconstruction; DLR = deep learning reconstruction; CE = contrast-enhanced; Likert-scale ranging from 1 - 5 with 5 being the best rating.

**Supplementary Table 2.** Median (interquartile range) and mean (standard deviation) ratings in conventionally reconstructed and deep learning reconstructed sequences for image quality and diagnostic confidence for pooled data for all raters.

|  | **CR** | |  | **DLR** | | **p-value** |
| --- | --- | --- | --- | --- | --- | --- |
|  | **Mdn (IQR)** | **M ± SD** |  | **Mdn (IQR)** | **M ± SD** |  |
| **Image quality** |  |  |  |  |  |  |
| **FLAIR** | 4 (4-4) | 4.07 ± 0.52 |  | 5 (5-5) | 4.79 ± 0.41 | < 0.001 |
| **T2** | 4 (4-4.75) | 4.18 ± 0.54 |  | 5 (5-5) | 4.84 ± 0.40 | < 0.001 |
| **T1CE** | 4 (4-4) | 3.89 ± 0.40 |  | 5 (5-5) | 4.79 ± 0.41 | < 0.001 |
| **Diagnostic confidence** |  |  |  |  |  |  |
| **FLAIR** | 4 (4-5) | 4.31 ± 0.65 |  | 4 (4-5) | 4.36 ± 0.63 | 0.132 |
| **T2** | 4 (4-5) | 4.22 ± 0.71 |  | 4 (4-5) | 4.28 ± 0.68 | 0.034 |
| **T1CE** | 4 (4-5) | 4.24 ± 0.61 |  | 4 (4-5) | 4.34 ± 0.66 | 0.012 |

The p-values were calculated using the Wilcoxon signed-rank test. CR = conventional reconstruction; DLR = deep learning reconstruction

; Mdn = median; IQR = interquartile range; M = mean; SD = standard deviation; FLAIR = Fluid-Attenuated Inversion Recovery; T2 = T2-weighted images; T1CE = T1-weighted contrast-enhanced images
